# A structural analog of ralfuranones and flavipesins promotes biofilm formation by *Vibrio cholerae*

**DOI:** 10.1371/journal.pone.0215273

**Published:** 2019-04-18

**Authors:** Mahtab Waseem, Jason Q. L. Williams, Arumugam Thangavel, Patrick C. Still, Patrick Ymele-Leki

**Affiliations:** 1 Department of Chemical Engineering, Howard University, Washington, District of Columbia, United States of America; 2 Department of Chemistry and Biochemistry, California State University, Dominguez Hills, Carson, California, United States of America; High Point University, UNITED STATES

## Abstract

Phosphoenolpyruvate-carbohydrate phosphotransferase system (PTS) is a highly conserved, multistep chemical process which uses phosphate transfer to regulate the intake and use of sugars and other carbohydrates by bacteria. In addition to controlling sugar uptake, the PTS regulates several bacterial cellular functions such as chemotaxis, glycogen metabolism, catabolite repression and biofilm formation. Previous studies have shown that the phosphoenolpyruvate (PEP) to pyruvate ratio is a critical determinant of PTS functions. This study shows that 2-oxo-4-phenyl-2,5-dihydro-3-furancarbonitrile (MW01), a compound with structural similarity to known natural products, induces *Vibrio cholerae* to grow preferentially in the biofilm mode in a mechanism that involves interaction with pyruvate. Spectrophotometric assays were used to monitor bacterial growth kinetics in microtiter plates and quantitatively evaluate biofilm formation in borosilicate glass tubes. Evidence of MW01 and pyruvate interactions was determined by nuclear magnetic resonance spectroscopy. Given the established connection between PTS activity and biofilm formation, this study also highlights the potential impact that small-molecule modulators of the PTS may have in the development of innovative approaches to manage desired and undesired microbial cultures in clinical, industrial and environmental settings.

## Introduction

Antimicrobial resistance against effective antibiotics is a global issue with societal, environmental, and economic repercussions [1, 2]. This global challenge is exacerbated by the ability of most bacteria to grow within structured microbial communities called biofilms. Made of single- or multi-species communities, biofilms enable microorganisms to withstand antibiotic dosages of up to 100–1000 times higher than they could resist as free-floating cells and to more rapidly develop resistance to antimicrobial mechanisms [3–8]. However, owing to the natural complexity of biological ecosystems, non-pathogenic biofilms are the mode of subsistence of select bacterial organisms in the environment–as is the case for *Vibrio cholerae*.

*Vibrio cholerae* is a halophilic, Gram-negative rod responsible for millions of cases of cholera each year [9, 10]. Current knowledge of the modes of growth of *V*. *cholerae* has shown that the organism naturally subsists within non-pathogenic biofilms in marine environments such as estuaries and oceans. However, its transition from the aquatic environment to human hosts correlates with significant changes in gene expression patterns and subsequent expression of virulence factors with dire consequences for the host [11]. Regulation of *V*. cholerae response to environmental changes and stress is controlled by metabolic pathways such as the bacterial phosphoenolpyruvate phosphotransferase system (PTS). The PTS is a multicomponent phosphor-transfer cascade that mediates transport and phosphorylation of selected sugars, such as glucose, sucrose, mannose and N-acetylglucosamine [12]. The PTS is also a highly conserved signal transduction cascade that integrates multiple independent pathways to regulate chemotaxis, glycogen catabolism, detection of quorum sensing molecules and biofilm formation [12–18].

**Fig 1:**
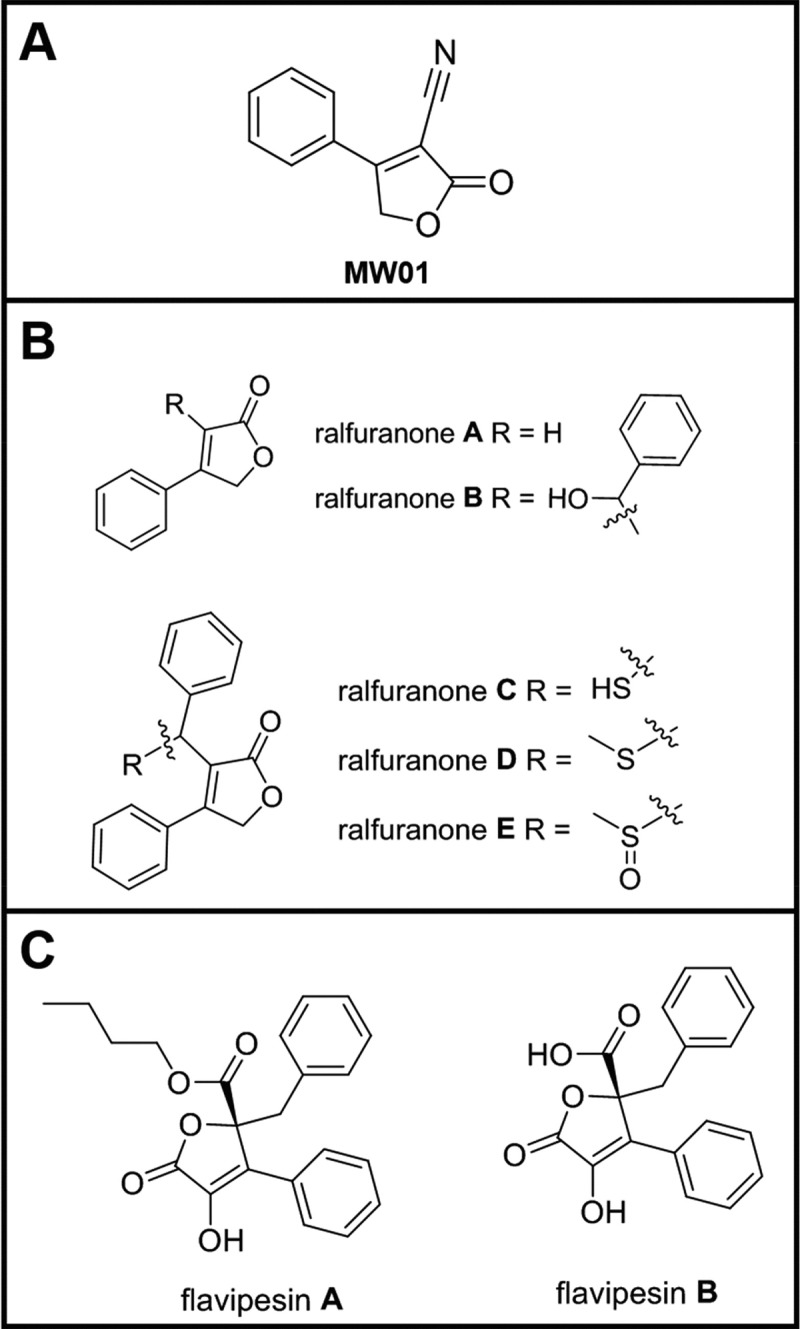
Synthetic compound 2-oxo-4-phenyl-2,5-dihydro-3-furancarbonitrile (MW01) has structural similarities with natural products. MW01 (A) is a synthetically produced compound with structural parallels to natural products, such as ralfuranones (B) and flavipesin (C).

A chemical screening assay was recently developed to identify small-molecule compounds that may interfere with a specific activity controlled by the PTS system, namely the uptake of PTS carbohydrates [19]. As previously described, a pilot assay was conducted in 384 well microtiter dishes using plates 1568 and 1569 from the Prestwick Collection, a commercial library, to validate the screen [19]. For this study, the commercial compounds used to develop the screening assay were reviewed for commercial availability and cross-referenced for structural similarity with other chemical compounds using SciFinder (CAS, Columbus, OH). One compound, 2-oxo-4-phenyl-2,5-dihydro-3-furancarbonitrile, labeled MW01 in this study, was singled out for its structural similarity to known natural products with antimicrobial properties (Fig 1). Owing to the reliance of the previously developed screen on PTS sugar transport and fermentation [19], this study investigated the hypothesis that MW01 could modulate bacterial PTS-functions. Results suggest a role for this compound in the regulation of bacterial biofilm formation by *V*. *cholerae* through interaction with pyruvate.

## Materials and methods

### Bacterial strains and media

A *V*. *cholerae* O139 strain MO10 (WT) served as the model organism for phosphoenolpyruvate phosphotransferase (PTS) activity and biofilm formation [5]. A *V*. *cholerae* PTS mutant (ΔEI), which is unable to transport sucrose and constitutively expresses enhanced biofilm formation, was used as a control [19, 20]. Bacterial glycerol stocks stored at –80°C were revived on Luria-Bertani (LB) agar plates.

As applicable, a previously described minimal medium (MM) was supplemented with different carbohydrates to test for bacterial fermentation, growth, and biofilm formation [19]. MM^Glu^ contained glucose (0.5% wt/vol), which is transported into *Vibrio* cells by the PTS cascade and other bacterial transport pathways, to assess bacterial growth. MM^Pyr^ contained pyruvate (0.5% wt/vol) to assess effects on biofilm formation. Additionally, MM supplemented with sucrose and pH indicators (pH-MM^Suc^) was used as a reporter medium to assess MW01 activity on sugar translocation and fermentation. pH-MM^Suc^ contained sucrose (0.5% wt/vol) in combination with thymol blue (0.006% wt/vol) and bromothymol blue (0.006% wt/vol), as pH indicators [19]. PTS is solely responsible for the transport of sucrose into *V*. *cholerae* cells [12, 21].

The chemical compound of interest, 2-oxo-4-phenyl-2,5-dihydro-3-furancarbonitrile (MW01; C_11_H_7_NO_2_; MW: 185.2; ID: 7D-136; CAS Registry Number: 7692-89-9), was purchased from Key Organics Inc (Bedford, MA). To investigate the activity of the compound against bacterial cultures, stock solutions of 15 mg/mL were prepared by suspending the powder in dimethyl sulfoxide (DMSO). When applicable, two-fold serial dilutions of the stock solutions were performed with DMSO to achieve working solutions at desirable concentrations of the compound. Control experiments were performed with DMSO alone to confirm that, as previously described [16, 19], DMSO did not interfere with subsequent carbohydrate translocation and biofilm formation assays.

### Sugar translocation and fermentation assay in microtiter plates

The activity of MW01 against phosphoenolpyruvate (PEP)-dependent sugar translocation and subsequent fermentation was assessed in pH-MM^Suc^ medium, as previously described [19]. Briefly, *V*. *cholerae* derived from a glycerol stock was streaked on an LB-agar plate and incubated overnight at 37°C. A loopful of cells was harvested, washed and resuspended in PBS at an optical density of 0.025 at 600 nm (OD_600_ = 0.025). For the assay, 30 μL of bacterial cell suspension was aliquoted into the wells of a 96-well plate containing 90 μL of pH-MM^Suc^ and 1 μL of working compound solution. Final concentrations of the compound in assay medium ranged from 2- to 124-μg/mL. For each assay, absorbance measurements at 615 nm (A_615_) were recorded every hour for a period of 24 hours. This step was automated using a Tecan Infinite M200 PRO microplate reader. The 96-well plates were continuously incubated at 30°C in the plate reader without shaking. As previously described, fermentation decreases the pH of the medium [19]. The pH indicators in the medium make it possible to monitor medium acidification spectrophotometrically through a change in absorbance at 615 nm. Thus, A_615_ in pH-MM^Suc^ primarily reflects the ability of cells to transport (and subsequently ferment) the sugar in the presence of the compound [19]. At least three experimental replicates were performed per assay plate, and each plate experiment was repeated multiple times. To account for the variability of initial absorbance measurements, experimental data were normalized to the initial A_615_ for each well. Results are reported as a mean measurement. Error bars represent the standard deviations, and statistical significance was calculated using a two-tailed *t* test. Measurements were considered significant if the *P* value was less than 0.05.

### Quantitative analysis of *V*. *cholerae* growth and biofilm formation in broth cultures

Quantification of *V*. *cholerae* biofilms in culture tubes was performed as previously described [12, 16]. Briefly, *V*. *cholerae* strains were grown overnight on LB agar plates at 27°C. To set up biofilm cultures, the resulting colonies were used to inoculate borosilicate tubes filled with 1 μL of working compound solution and 300 μl of MM^Pyr^ or LB broth. The starting inoculum of *Vibrio* cells had an optical density of 0.05 at 600 nm (OD_600_). After incubation for approximately 20 hours for MM^Pyr^ or 18 hours for LB broth at 27°C, planktonic cell suspensions were collected, to leave only surface attached cells remaining in each tube. The planktonic cell density was determined by measuring the optical density at OD_600_. To quantify biofilm formation, 300 μl of PBS and a small volume of 1-mm-diameter glass beads (Biospec, Inc.) were added to the surface-attached cells remaining in the borosilicate tube, and the cells were dispersed by vortexing. The amount of biofilm formed for each culture was determined by measuring the optical density at OD_600_ of the resulting cell suspension using a Tecan Infinite M200 PRO microplate reader. The reported total growth was calculated from the sum of the OD_600_ values measured for planktonic and surface-associated cell suspensions. All values represent the means of the results of at least three experimental replicates. Error bars represent the standard deviations and statistical significance calculated using a two-tailed *t-*test. Differences were considered significant if the *P* value was less than 0.05.

### Quantitative determination of cell viability on agar plates

Similarly to the setup of biofilm assays in borosilicate tubes, *V*. *cholerae* cells from frozen glycerol stocks were revived overnight on LB agar plates at 37°C. The resulting colonies were used to inoculate borosilicate tubes filled with 1 μL of working compound solution and 300 μl of LB broth. After incubation for approximately 4 hours at 27°C, all cultures were subjected to an identical series of ten-fold dilutions. Then 10 μL of broth solutions were seeded onto LB agar plates and cultured overnight at 37°C. The number of colony-forming units (CFUs) formed on each plate was determined with a Heathrow Scientific eCount colony counter. Conditions were tested in triplicate in each experiment (that is, three culture tubes were inoculated for each tested condition and three separate plates were seeded per culture tube). Three experimental replicates were performed on separate days.

### Evidence of chemical reaction between MW01 and pyruvate or PTS-carbohydrates

Chemical reactivity between MW01 and pyruvate or PTS-carbohydrates was tested by incubating 100-μg/mL of MW01 in minimal media supplemented with pyruvate or the carbohydrates. Following the protocol of *V*. *cholerae* biofilm assays, 1 μL of working compound solution was mixed in borosilicate tubes with 300 μl of DI water, a carbohydrate (0.5% wt/vol) solution in water, an amino-acid solution, and a phosphate buffer solution. The carbohydrates tested included mannose, fructose, glucose, and sucrose. The amino-acid and the phosphate buffer solutions were prepared in similar concentrations to those present in minimal media [19]. After incubation for 18 to 24 h at 27°C, the tubes were visually monitored for a possible color change.

### Evidence of chemical reaction between MW01 and growth media

Reactivity of MW01 (**1**) and media components was accessed by replicating the biological growth conditions, while maintaining concentrations of MW01 high enough to be observed by NMR spectroscopy. MW01 (Key Organics, 5 mg) was dissolved in 200 μL DMSO-d_6_ and diluted by slow addition of 200uL of a 0.5 w/v% pyruvic acid solution, in a 4mL glass scintillation tube (Fisher Scientific). The reaction was capped and left to react for 12 hours at room temperature on the lab bench. A brilliant blue pigment was formed as seen in the biological media preparations and the reaction mixture was transferred directly to a 5mm NMR tube and proton spectra obtained.

## Results

### MW01 has a structural similarly with known bioactive natural products

The structure of MW01 (**1**) is similar to two previously characterized natural products also bearing biofilm modulatory activity: the ralfuranones and flavipesins (Fig 1).

Ralfuranones are aryl-furanone secondary metabolites originally isolated by Schneider and coworkers that are extracellularly secreted by a soil-borne bacterium, *Ralstonia solanacearum* [22, 23]. Biofilm expression and pathogenicity of *R*. *solanacearum* are controlled by complex regulatory networks that respond to environmental conditions through quorum sensing mechanisms [24]. Ralfuranones have been shown to contribute to the virulence of *Ralstonia solanearum* by influencing quorum-sensing signaling pathways [24, 25].

Flavipesins are butenolide natural products originally isolated from a marine-derived endophytic fungus, *Aspergillus flavipes* AIL8 [26] by Bai and coworkers. The flavipesins differ from MW01 at C5, at which they have a phenyl and ester substituent, instead of a nitrile (Fig 1). Flavipesin A exhibited modest antimicrobial activity against two Gram-positive pathogens, *Staphylococcus aureus* and *Bacillus subtilis*, as well as antibiofilm activity against mature *S*. *aureus* biofilms [26].

Given its structural similarities to known bioactive natural products, we hypothesized that MW01, which showed preliminary activity in a cell-based assay that relied on fermentation of a PTS-sugar [19], may interfere with PTS functions.

### MW01 impedes *V*. *cholerae* sugar fermentation

**Fig 2:**
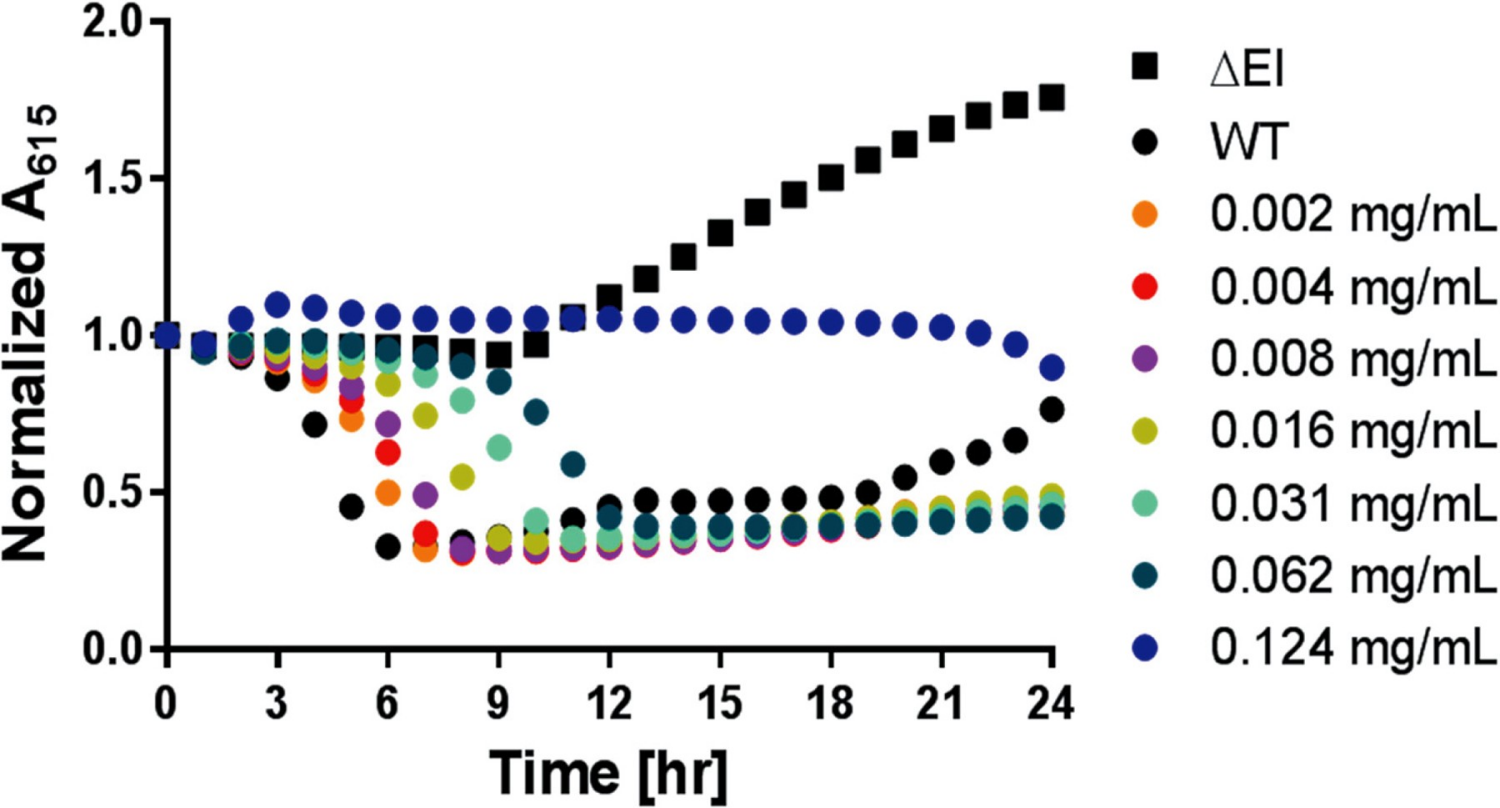
Impact of MW01 on sucrose fermentation by *V*. *cholerae*. Assays were carried out at 30°C in pH-MM^Suc^ and microtiter plates. Absorbance measurements (A_615_) reflect the ability of bacterial cells to transport and ferment sucrose. The result of one representative experiment is shown. Data are shown for a PTS mutant (ΔEI) negative control, a wild-type *V*. *cholerae* (WT) positive control, and WT in the presence of the compound. Bacteria were exposed to MW01 at concentrations varying from 0.002- to 0.124-mg/mL. Conditions were tested in triplicate in each experiment, and three experimental replicates were performed on separate days. To account for the variability of initial absorbance measurements, experimental data were normalized to the initial A_615_ for each well.

A previously established high-throughput screen to identify natural product extracts with anti-bacterial activity resulted in the characterization of a novel broad spectrum antimicrobial agent [19]. The assay uses *V*. *cholerae* as a model pathogen to probe anti-microbial properties of candidate compounds such as MW01, based on spectrophotometric assessments of sugar fermentation, which can only occur in viable bacteria [19]. Medium acidification was monitored with MW01 concentrations ranging from 2 to 124 μg/mL in a minimal medium supplemented with sucrose and pH indicators (pH-MM^Suc^). PTS is solely responsible for the transport of sucrose into *V*. *cholerae* cells. Thus, pH-MM^Suc^ was used as a reporter medium to assess MW01 activity on sugar translocation and fermentation [12, 21]. Conditions were tested in triplicate for each experiment and two experimental replicates were performed on separate days. Within the range of concentrations investigated, MW01 inhibited medium acidification in a concentration dependent manner (Fig 2). Bacterial activity was completely inhibited at 124 μg/ml, the highest concentration tested.

### MW01 interferes with growth rates and modes of growth of V. cholerae

**Fig 3:**
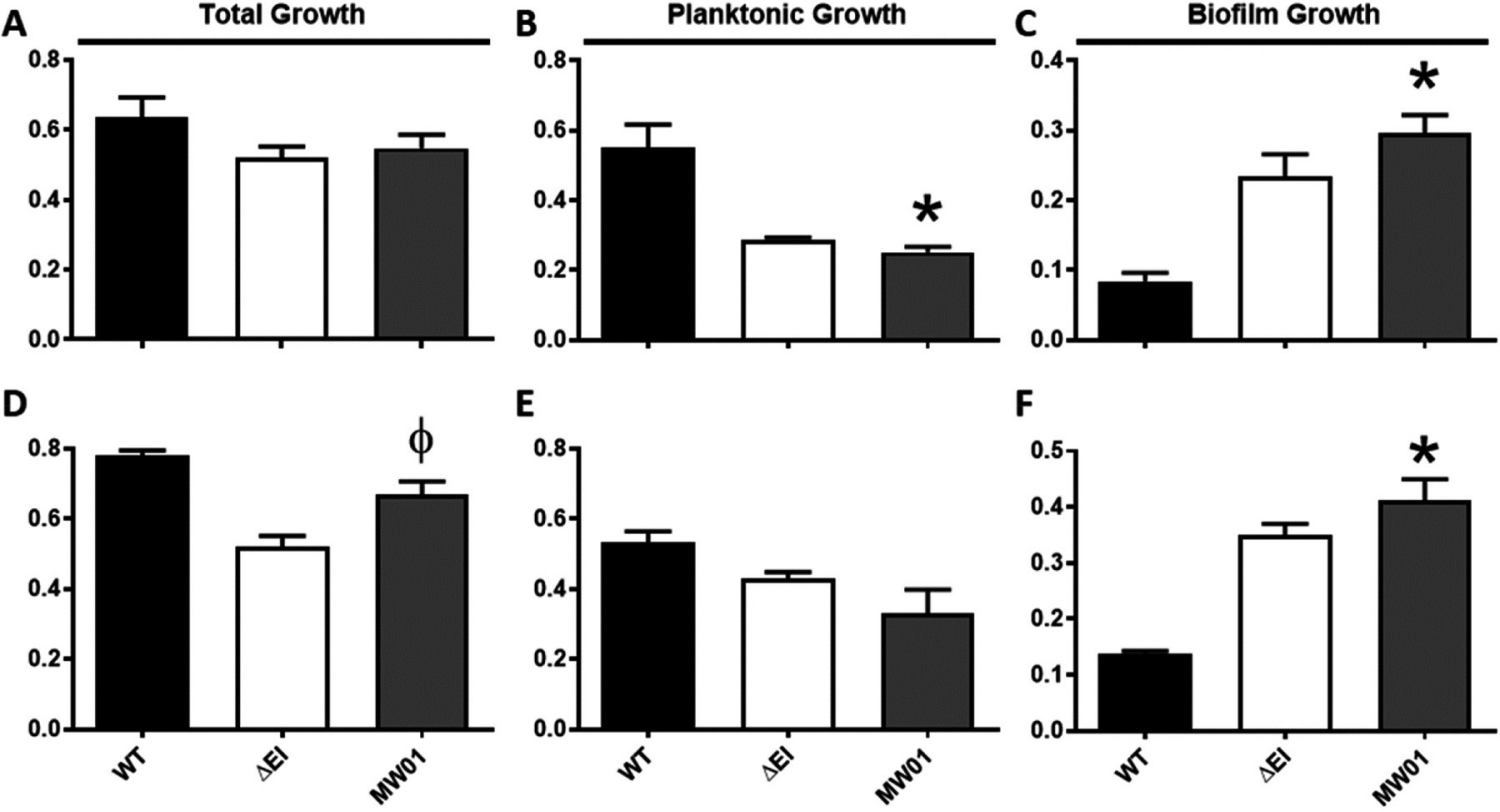
MW01 induces *V*. *cholerae* biofilm formation without effect on total growth in LB. *V*. *cholerae* cultures were grown in borosilicate tubes in LB broth supplemented with 0.2 mg/mL of MW01 (A–C) or 0.1 mg/mL of MW01 (D–F). After 18 hours of incubation at 27°C, biofilm cells were harvested and quantified for total growth (A and D), planktonic growth (B and E), and biofilm growth (C and F). Data are shown for a PTS mutant (ΔEI) positive control growth in LB, wild-type *V*. *cholerae* (WT) in LB alone, and WT in LB supplemented with the compound. * and Ø denotes statistical significance *(p < 0*.*05)* from WT and ΔEI control values respectively.

To determine whether MW01 manifested bacteriostatic or bactericidal properties at 124 μg/ml, *Vibrio* cells were exposed to MW01 at this concentration in broth and agar cultures. First, broth assays in bacterial culture tubes were performed with MW01 at concentrations of 100- and 200 μg/mL. *V*. *cholerae* cell suspensions supplemented with MW01 were inoculated in borosilicate tubes for 18 hours at 27°C in LB broth. The cells exposed to MW01 were able to growth to density levels that were slightly lower than wildtype (WT) controls but greater than PTS mutant (ΔEI) controls (Fig 3). ΔEI and other PTS-components mutants are known to display slightly reduced growth compared to wildtype, likely due to the lack of activity of the PTS system and the reduced uptake of PTS-dependent carbohydrates present in the growth medium [20, 27]. The inactivity of PTS components in ΔEI is known to be associated with significant increases in biofilm formation [12, 20, 21], and was corroborated here(Fig 3). A significant increase in biofilm formation was recorded with *Vibrio* cultures challenged with MW01. The compound induced biofilm formation up to threefold when compared to WT controls and to levels similar to that of biofilm producing ΔEI controls (Fig 3).

**Fig 4:**
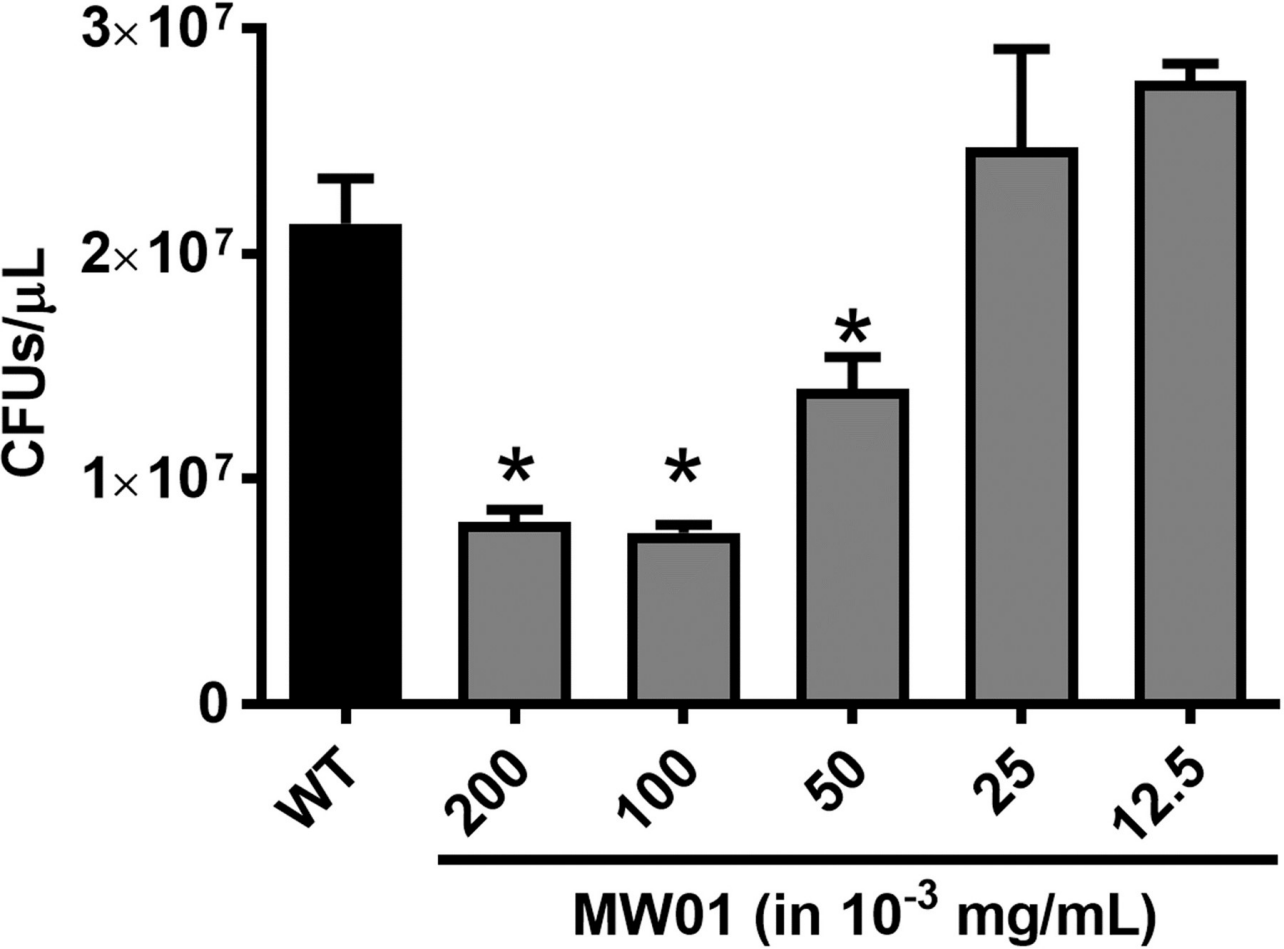
MW01 impedes growth of *V*. *cholerae* in early stages (within the first four hours) of bacterial culture. *V*. *cholerae* cells were first exposed to MW01 in LB broth for four hours at 27°C in a setup that mimicked the initiation of biofilm cultures in borosilicate tubes. Then, after identical series of ten-fold dilutions, 10 μL of each broth culture was plated on LB agar and incubated overnight at 37°C. Wild-type *V*. *cholerae* (WT) not exposed to MW01 served as control. * denotes statistical significance *(p < 0*.*05)* from control values.

Additionally, agar assays were performed with cells harvested at earlier time points (within four hours of initiation) of the bacterial cultures to monitor the effect of MW01 on bacterial growth prior to the initiation of biofilm development. Samples of cells exposed to MW01 in LB broth for four hours were plated on LB agar. The reduced incubation period in LB broth prevented innate bacterial biofilm formation by *V*. *cholerae*. In these conditions, MW01 inhibited the growth of *V*. *cholerae* planktonic cells (Fig 4). Exposure to MW01 at concentrations of 100- and 200 μg/mL resulted in a three-fold and ten-fold reduction in colony forming units (CFUs), respectively, when compared to control values. These data confirmed that MW01 specifically impedes the planktonic mode of growth of *V*. *cholerae* cells.

**Fig 5:**
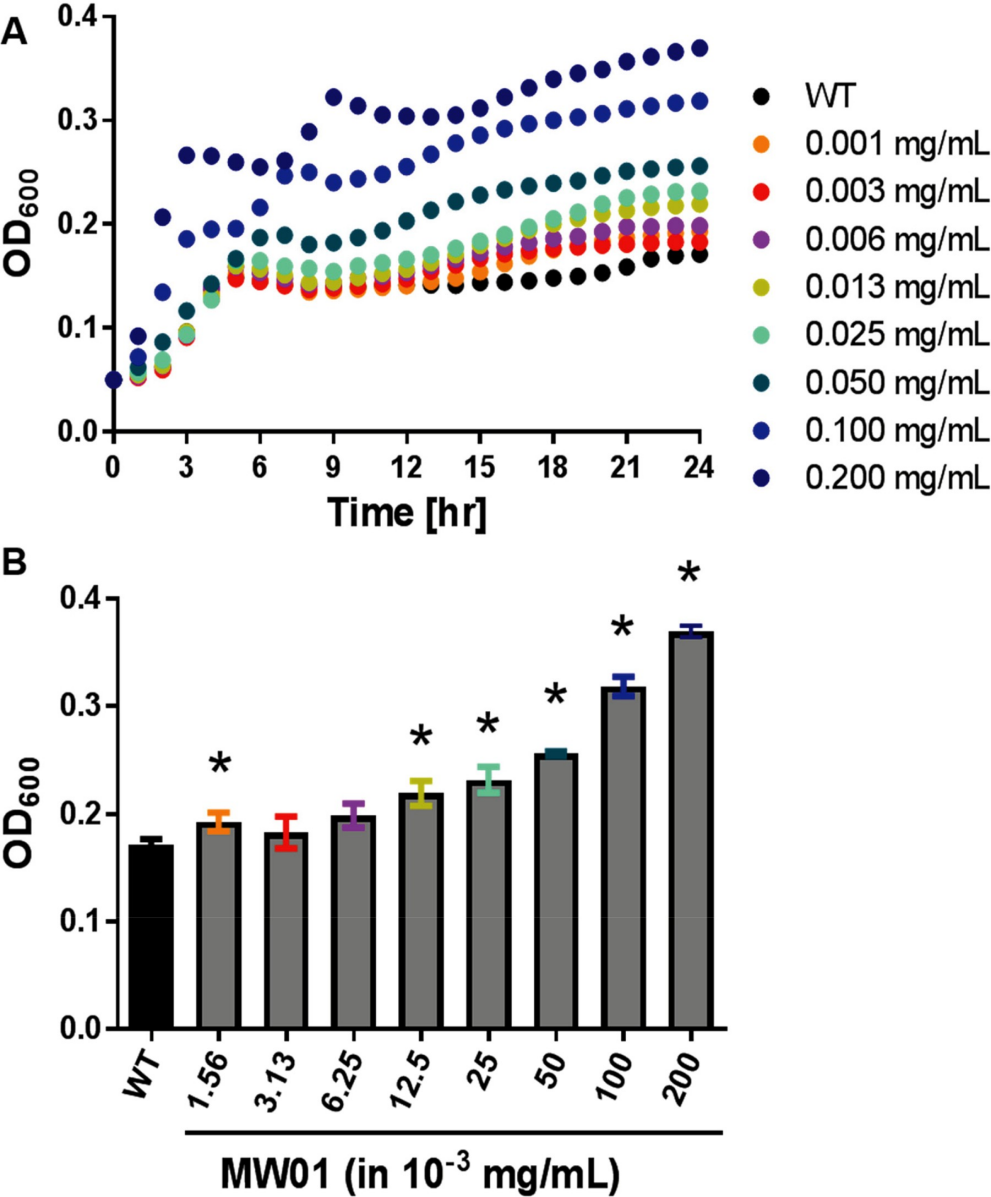
Impact of MW01 on *V*. *cholerae* growth in MM^Glu^. Assays were carried out at 30°C in microtiter plates. Optical density measurements at 600 nm (OD_600_) reflect the ability of *V*. *cholerae* to grow (both as planktonic and biofilm cells) in minimal medium supplemented with 0.5% wt/vol glucose (MM^Glu^). Data are shown for a wild-type *V*. *cholerae* (WT) alone or in the presence of MW01, as (A) bacteria grow over time in 96-well plate cultures and (B) at the 24-hour mark of the growth culture. Bacteria were exposed to MW01 at concentrations varying from 0.001- to 0.200-mg/mL. Conditions were tested in triplicate in each experiment, and three experimental replicates were performed on separate days.

To investigate the possibility that MW01 may impede planktonic growth of *V*. *cholerae* without negatively affecting total growth by this pathogen, challenge assays with MW01 were performed in minimal medium supplemented with 0.5% (wt/vol) glucose (MM^Glu^). Unlike sucrose which is only transported into *V*. *cholerae* cells by the multicomponent PTS cascade, glucose can be facultatively transported by the PTS cascade or by an alternative transport system (Fig 2) [21]. Furthermore, addition or presence of glucose in minimal and LB growth media enable the formation of bacterial biofilms by *V*. *cholerae* cells through multiple (PTS-dependent and PTS-independent) pathways [12, 18]. In MM^Glu^, MW01 appeared to significantly enhance the growth rate of *Vibrio* cells (Fig 5B). This resulted in greater total bacterial counts after 24 hours of incubation in culture plates in the presence of MW01, at concentrations greater than 6.25 μg/mL (Fig 5B). Taken together these results suggested that MW01 impedes planktonic growth of *V*. *cholerae* but does not possess bactericidal properties at concentrations as high as 200 μg/mL. Glucose is known to enhance growth and biofilm formation in *V*. *cholerae* when the PTS is inactivated [20]. It is likely that MW01 promoted the biofilm mode of growth of *V*. *cholerae* cells–and that the higher apparent bacterial yield counts in Fig 5 were representative of forming bacterial biofilms which resulted in greater absorbances and higher optical density values.

### MW01 induces V. cholerae biofilm formation in a concentration dependent manner

**Fig 6:**
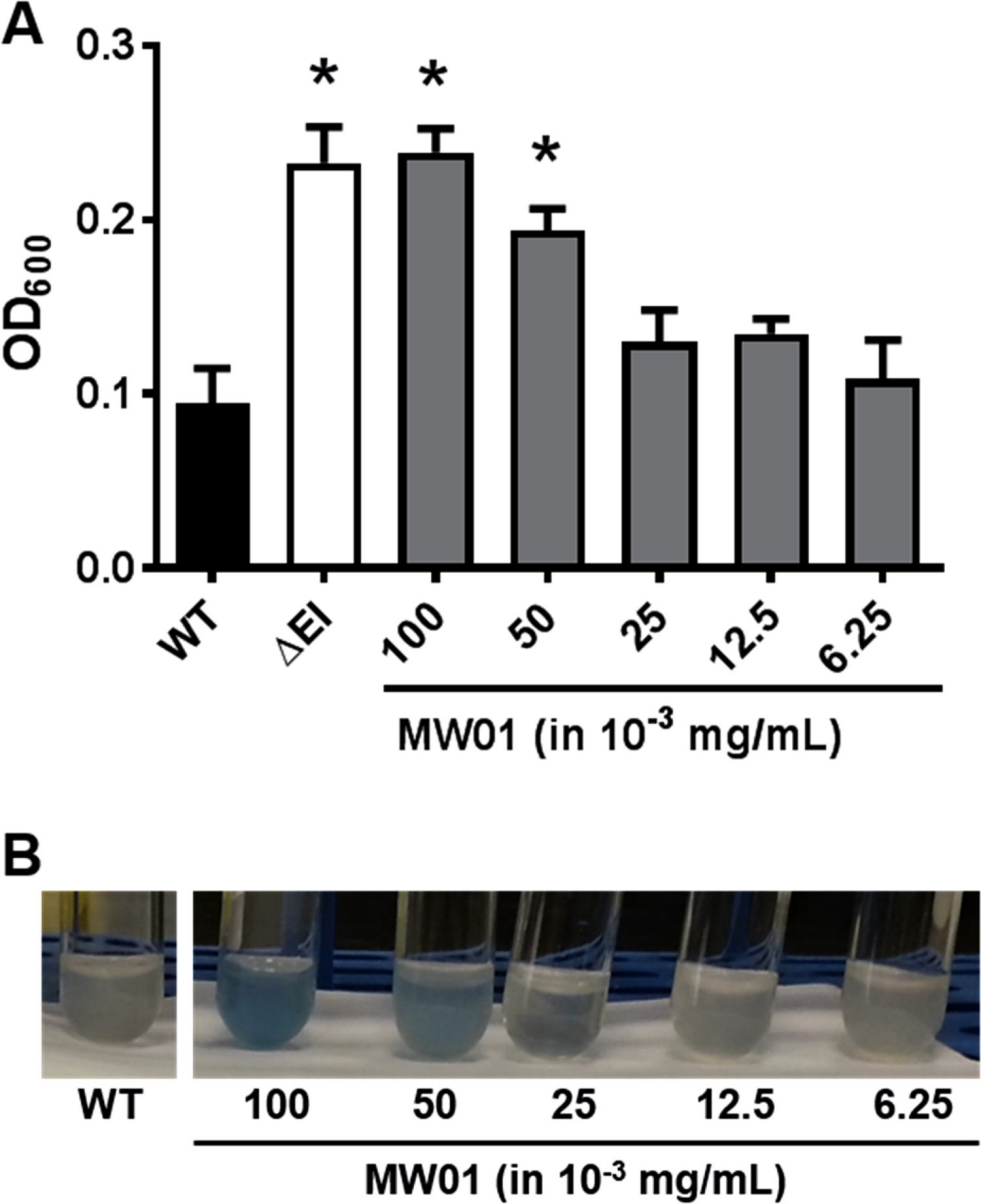
Impact of MW01 concentration on *V*. *cholerae* biofilm formation. (A) Wild type (WT) *V*. *cholerae* cultures were used to inoculate borosilicate tubes filled with 1 μL of working MW01 solutions, at concentrations ranging from 0.00625–0.1 mg/mL, and 300 μl of MM^Pyr^ broth. The resulting biofilm cells were harvested after 20 hours of incubation at 27°C, and their optical densities were determined at 600 nm (OD_600_). A PTS mutant (ΔEI) served as positive control for biofilm growth. Stars indicate measurements that were statistically significantly different from WT biofilm formation in MM^Pyr^ alone. (B) WT *V*. *cholerae* biofilms forming at the air-liquid interface in borosilicate culture tubes while growth medium changes coloration in presence of MW01.

Pyruvate is not transported by the PTS. MM supplemented with pyruvate (MM^Pyr^) is commonly used to examine the role of PTS-dependent regulation of *V*. *cholerae* biofilm formation in the absence of effects on bacterial growth [12, 19, 21]. To further evaluate the role of MW01 on *Vibrio* biofilm formation, different concentrations of MW01 were added to MM^Pyr^. A *V*. *cholerae* mutant, ΔEI, which constitutively expresses enhanced biofilm formation, was used as a control [19, 20]. Prior studies in MM^Pyr^ demonstrated that a deletion of the PTS Enzyme I (EI) promotes *V*. *cholerae* biofilm growth, because phosphorylation of specific PTS proteins is necessary for the repression of surface-associated growth [12, 20]. Data suggest that, in MM^Pyr^, MW01 at concentrations as low as 0.05 mg/mL induces biofilm formation at levels comparable to those of ΔEI (Fig 6A). However, these experiments revealed a color change of the culture media, which turned blue in the presence of MW01 (Fig 6B), indicative of a chemical reaction between MW01 and constituents of MM^Pyr^.

### Evidence of pyruvate-catalyzed “keto-enol” tautomerization of MW01

**Fig 7:**
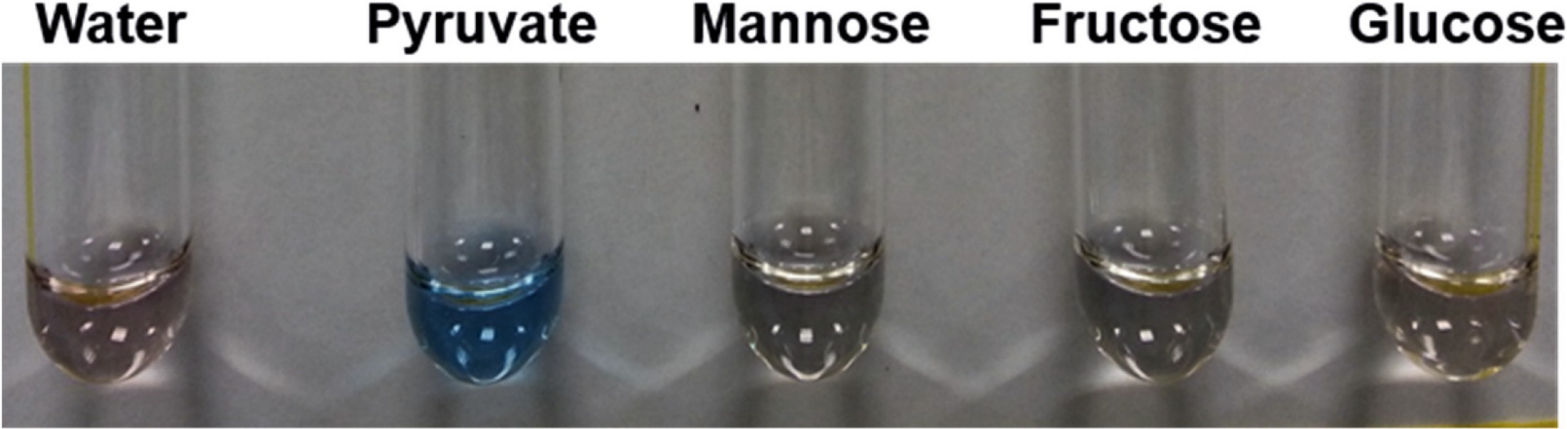
MW01 reacts with pyruvate to form a distinctive blue colored product. MW01 (0.1 mg/mL) in water and 0.5 wt/vol % aqueous solutions pyruvate and carbohydrates (mannose, fructose, and glucose).

The composition of minimal medium (MM) used in studies involving the role of PTS in regulation of growth of *V*. *cholerae* biofilms is well established [20]. Each of the salts and amino acids that constitute MM were tested at concentrations routinely used in *Vibrio cholerae* growth media with MW01. None yielding a color change to brilliant blue as observed in Fig 6 when *Vibrio* biofilms were challenged with MW01 in MM^Pyr^ (Data not shown). Further assays were performed with pyruvate and selected PTS sugars. Only the mixture of MW01 and pyruvate yielded a brilliant blue pigmentation similar to that observed in MM^Pyr^ during biofilm assays (Fig 7).

**Fig 8:**
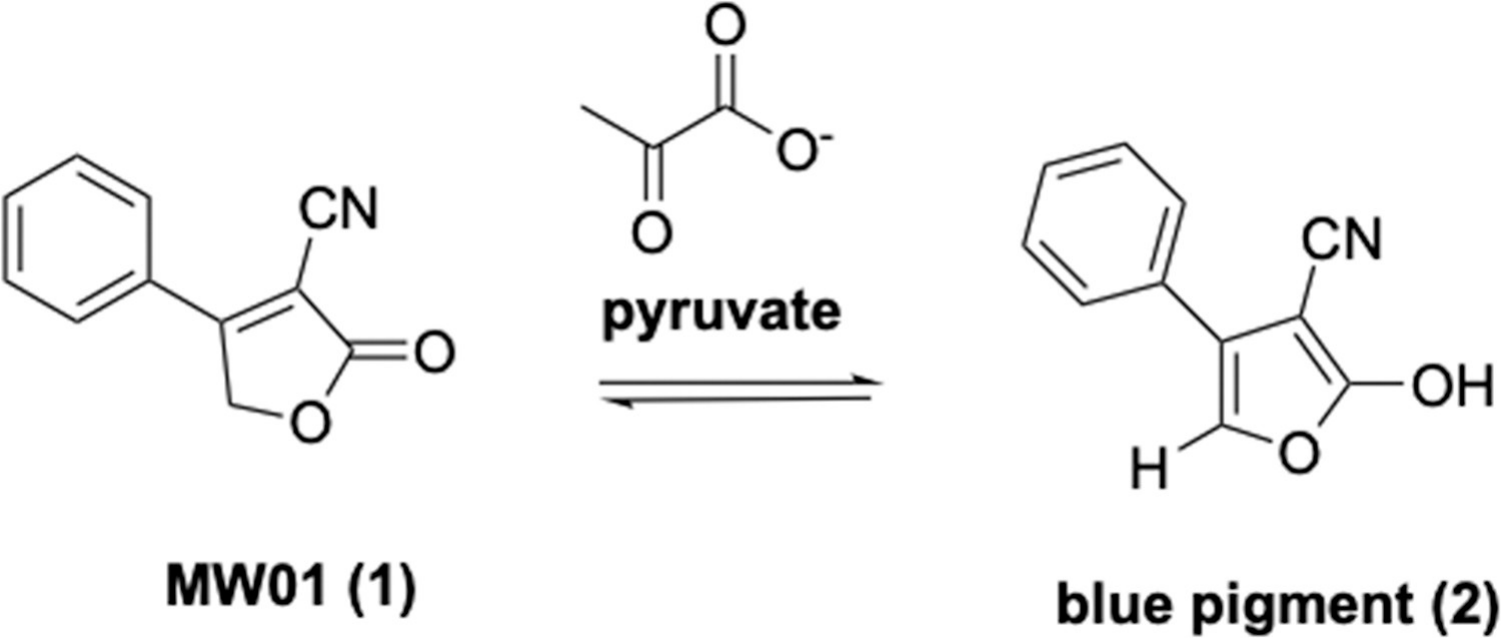
Proposed scheme showing the catalytic role of pyruvate in the reaction with MW01 (1). Only in the presence of pyruvate does the methylene (δ 5.71, 2H, s) undergo abstraction resulting in proposed blue pigment **2**.

NMR studies were used to further probe the chemical reaction responsible for the blue media pigmentation when *Vibrio* biofilms were challenged with MW01 (**1**) and pyruvate. In the absence of pyruvate, no blue pigmentation is observed. Rather, pyruvate is needed for the color change. To support the involvement of pyruvate in abstracting the methylene protons (δ 5.71, 2H, s) of MW01, a series of NMR experiments measuring the pyruvate methyl chemical shift in 1:1 DMSO-d_6_/D_2_0 were carried out (Panel A in S1 Fig). A 0.0147 ppm change of the pyruvate methyl group (δ 2.41, 3H, s) was observed after reaction with **1**. The distance between the reactive center carboxylate group and the methyl protons of pyruvate translate to minute changes in the proton NMR chemical shifts. The change in methyl group chemical shift along with disappearance of the methylene protons (δ 5.71, 2H, s) of MW01 after reaction with pyruvate, support pyruvate reaction with MW01 (Panel B in S1 Fig). Fig 8 shows a proposed scheme of the catalytic role of pyruvate in the reaction with compound **1**. The acidity of the methylene protons of similar compounds to **1** have been shown to result in furan tautomerism without the presence of base [28, 29]. However, this tautomeric equilibrium is not observed with MW01. Only in the presence of pyruvate does the methylene (δ 5.71, 2H, s) undergo abstraction resulting in proposed blue pigment **2** (Fig 8). Compound **2**, although not isolated and fully characterized from the complex reaction product mixture was detected in the ESI-MS nominal mass spectrum at *m/z* = 184.00 (S2 Fig). Blue pigment **2** rapidly degrades to green and gold pigments and full characterization will be the subject of follow-up studies.

## Discussion

Biofilm formation by *V*. *cholerae* is primarily dependent on two factors: presence of environmental calcium ions in concentrations comparable to those found in seawater, or production of *V*. *cholerae* exopolysaccharide (VPS) [17, 30, 31]. A paradigm for the control of biofilm formation by *V*. *cholerae* relies on control of the expression of the *vps* operon, which itself transcriptionally regulates the production of VPS polysaccharides by the phosphoenolpyruvate phosphotransferase system (PTS) [12, 17, 18, 32]. The PTS is highly conserved among bacterial organisms. It is a multicomponent signal transduction cascade that regulates chemotaxis, glycogen catabolism, detection of quorum sensing molecules, and bacteria biofilm formation, through multiple independent pathways [12–18]. In the lesser understood pathway, two components of the PTS^Ntr^ secondary system, EIIA^Ntr1^ and EIIA^Ntr2^, inhibit *vps* expression and *V*. *cholerae* biofilm formation [12, 18]. In a second pathway, glucose-specific PTS components in rich medium, EIIA^Glc^ and EIIBC^Glc^ may respectively activate and inhibit *vps* transcription under the influence of the transcriptional repressor Mlc [12, 18]. In a third pathway observed in minimal or rich growth media, phosphoenolpyruvate (PEP)-dependent, phosphorylated HPr or FPr inhibits *vps* expression and consequently inhibits biofilm formation by *V*. *cholerae* [12, 18].

**Fig 9:**
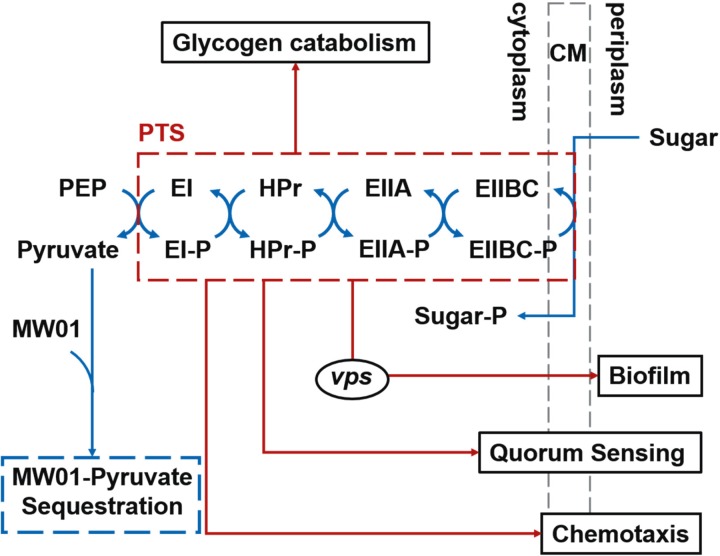
Schematic diagram of the PTS cascade. MW01 interaction with pyruvate, upstream of the phosphor-transferase cascade, would likely impact the regulation of *Vibrio* biofilm formation and other bacterial regulatory networks through variation of the PEP:pyruvate ratio and modification of the phosphorylation state of the PTS enzymes. This hypothetical scenario however does not preclude the possibility of the pyruvate-MW01 complex forming in media solution and inducing biofilm formation through yet undetermined interactions with *Vibrio* cells.

As stated in earlier studies, the phosphorylation state of PTS components reflects the intracellular availability of PEP and the environmental availability of PTS-specific sugars. PTS components become dephosphorylated when PEP is scarce and/or PTS-specific sugars are plentiful; they are phosphorylated when PEP is plentiful and transported sugars are scarce [16, 33]. Meanwhile, transport of PTS-carbohydrates into a bacteria cell is highly dependent on the PEP to pyruvate ratio of the cell [27, 34]. When *Vibrio* cells are grown in rich medium, containing PTS-carbohydrates, pyruvate sequestration by MW01 at the level of the PTS likely results in variations of the PEP:pyruvate ratio and mimics mutation scenarios in which EI and other early components of the PTS are inactivated (Fig 9). This situation also mimics environmental scenarios in which PEP is scarce and PTS-carbohydrate are abundant, resulting in the predominance of PTS components in a dephosphorylated state. These scenarios all lead to a relief of the inhibitory roles of HPr and FPr on *vps* expression and result in increased biofilm formation.

MW01 may form complex intermediary structures with pyruvate inhibiting the PTS-dependent fermentation properties of *Vibrio* cells; but it does not impede *Vibrio* growth in MM^Pyr^ in the absence of PTS-carbon sources; and it induces preference for biofilm growth–versus planktonic growth–in all culture conditions. Taken together, these results support the hypothesis that MW01 interacts with PTS elements upstream of the phosphotransferase cascade, through interaction with pyruvate (Fig 9). However, they do not preclude the possibility that MW01 promotes biofilm formation by *V*. *cholerae* through other pathways. The phosphorylation cascade that begins with PEP, within the PTS, regulates the translocation of various sugars and carbohydrates into the cell but also carries out regulatory functions related to carbon, nitrogen, and phosphate metabolism, as well as to potassium transport, chemotaxis, and bacterial virulence [35, 36]. Interference with the PTS by MW01, at the initial stage of the phosphotransferase cascade, may have both direct and indirect downstream consequences in the complex regulatory network of the microbial metabolism. It is also possible that the pyruvate-MW01 complex forms in media solution and induces biofilm formation through yet undetermined interactions with *Vibrio* cells. Thus, the elucidation of the mechanism(s) of action of MW01 through direct interaction with the biofilm-controlling properties of the PTS or indirect induction of biofilm formation in response to adaptive stress response by bacteria remains to be determined.

Additionally, the findings of this study warrant further microbiological and chemical studies of the potential biological and environmental impact of MW01 and similar compounds, that can modulate the functions of the PTS cascade. In the broader scope of applications to human disease, the possibility of using MW01 and similar compounds to control biofilm expression of *V*. *cholerae*; a mode of growth associated with attenuated virulence properties responsible for cholera outbreaks and epidemics, holds promise. Other studies have suggested that *Vibrio* biofilms could be re-engineered to serve as reservoirs for surface-active secreted proteins of biomedical, bioengineering, or biotechnological importance [37]. The fact that several bioactive natural products, including ralfuranones and flavipesins [22, 24, 25], contain structural similarities to MW01 and have demonstrated a shared propensity for interfering with the expression of bacterial biofilms further emphasizes the relevance of this naturally-occurring mode of control. Lessons learned from microbial and chemical ecology should guide future scientific endeavors, as it pertains to characterizing, building and managing chemical antimicrobial agents.

## Supporting information

S1 Fig Pyruvate catalyzes the reaction with MW01. (Panel A) 400 MHz proton NMR spectrum of MW01 in DMSO-d_6_. (Panel B) 400 MHz proton NMR spectrum of reaction product (upper spectrum), and pyruvate alone (lower spectrum) in 1:1 DMSO-d_6_/D_2_O. As shown in the inset spectrum, a 0.0147 ppm change of the pyruvate methyl group (δ 2.41, 3H, s) was observed after reaction with MW-01. The distance between the reactive center carboxylate group and the methyl protons of pyruvate translate to minute changes in the proton NMR chemical shifts. The change in methyl group chemical shift along with disappearance of the methylene protons (δ 5.71, 2H, s) of MW01 after reaction with pyruvate, support pyruvate reaction with MW01.
(DOCX)

S2 Fig Detection of blue pigment (2) by mass spectrometry. ESI-MS negative mode analysis of the reaction product between MW-01 and pyruvate showing a key signal at *m/z* = 184.00 (calculated: 184.04).
(DOCX)
