# Supplementary figures and images for: A structural analog of ralfuranones and flavipesins promotes biofilm formation by Vibrio cholerae
Source: PLoS One. 2019 Apr 18;14(4):e0215273. doi: 10.1371/journal.pone.0215273 (PMC6472748; doi:10.1371/journal.pone.0215273)

**Supporting Information S1**


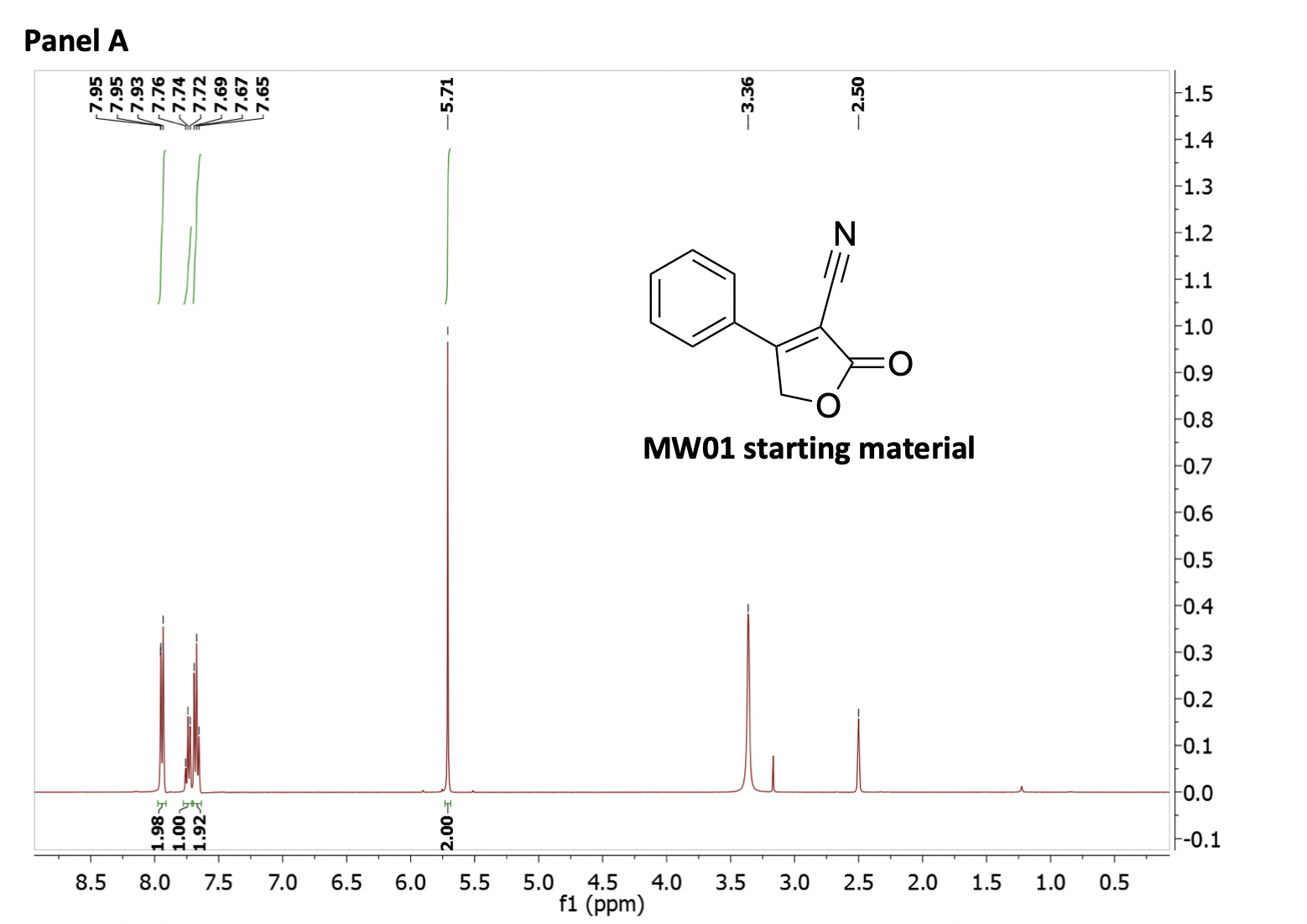


**Panel B**


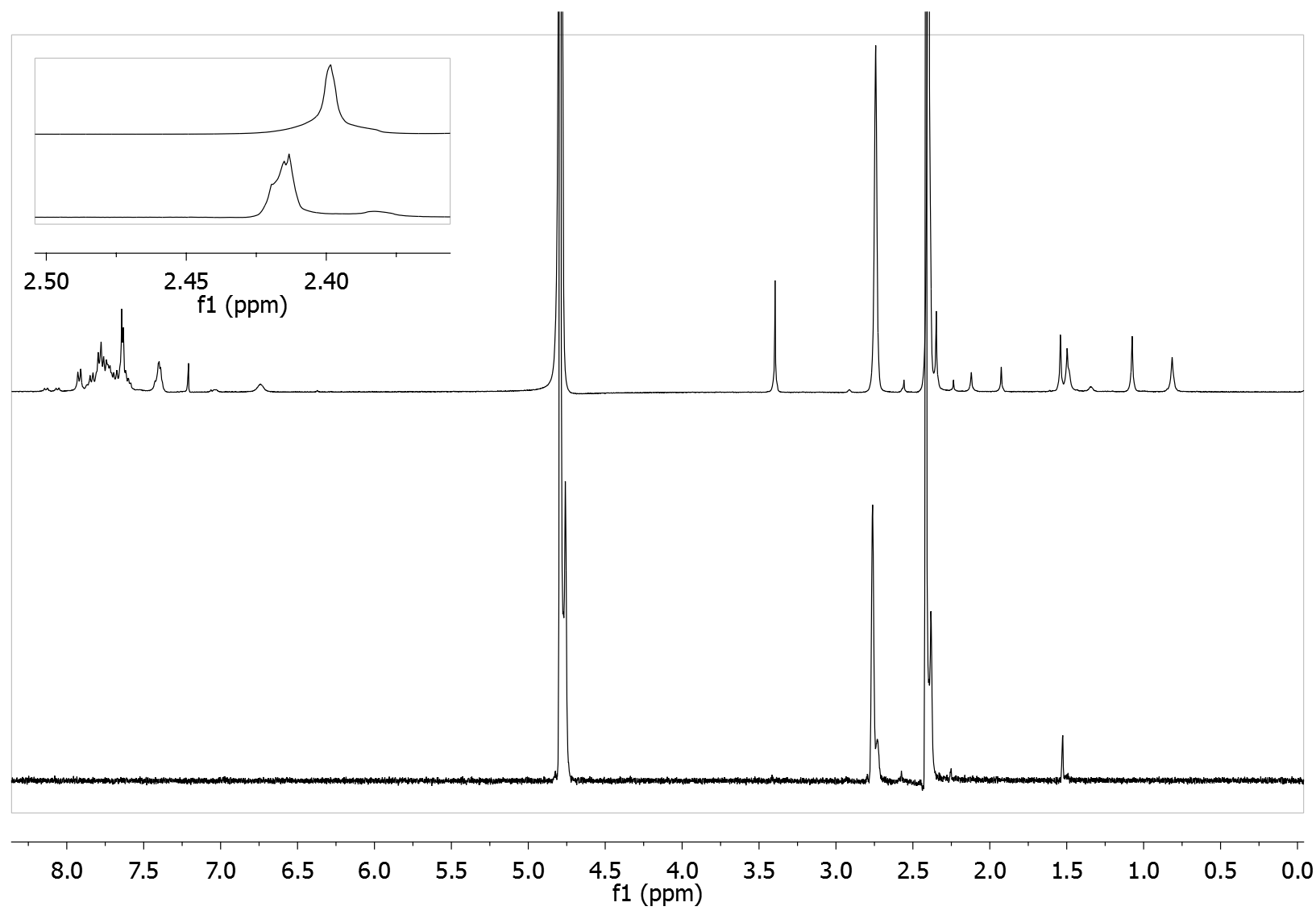

Supplement: S1 Fig — (Panel A) 400 MHz proton NMR spectrum of MW01 in DMSO-d6. (Panel B) 400 MHz proton NMR spectrum of reaction product (upper spectrum), and pyruvate alone (lower spectrum) in 1:1 DMSO-d6/D2O. As shown in the inset spectrum, a 0.0147 ppm change of the pyruvate methyl group (δ 2.41, 3H, s) was observed after reaction with MW-01. The distance between the reactive center carboxylate group and the methyl protons of pyruvate translate to minute changes in the proton NMR chemical shifts. The change in methyl group chemical shift along with disappearance of the methylene protons (δ 5.71, 2H, s) of MW01 after reaction with pyruvate, support pyruvate reaction with MW01. (DOCX) [file pone.0215273.s001.docx]

**Supporting Information S2**


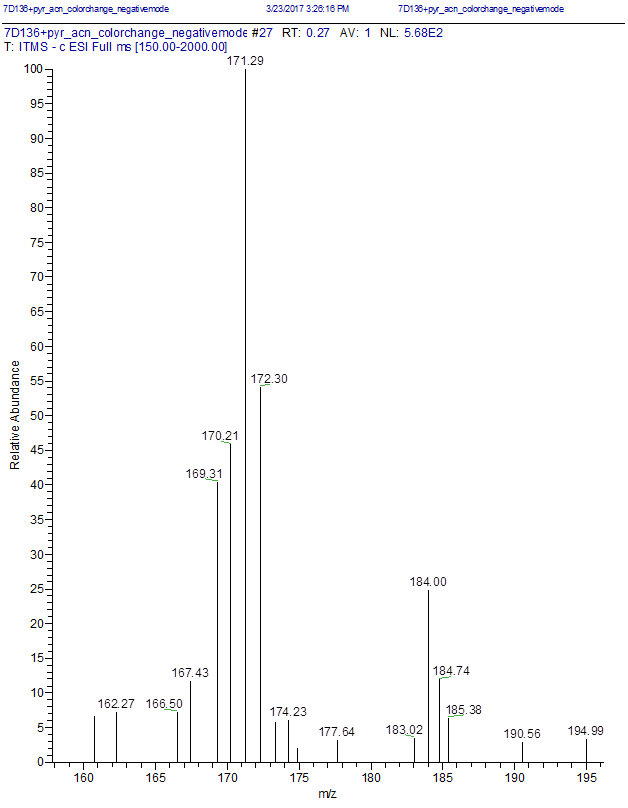

Supplement: S2 Fig — ESI-MS negative mode analysis of the reaction product between MW-01 and pyruvate showing a key signal at m/z = 184.00 (calculated: 184.04). (DOCX) [file pone.0215273.s002.docx]
